# Forecasting worldwide empty container availability with machine learning techniques

**DOI:** 10.1186/s41072-022-00120-x

**Published:** 2022-07-27

**Authors:** Christoph Martius, Lutz Kretschmann, Miriam Zacharias, Carlos Jahn, Ole John

**Affiliations:** 1 Fraunhofer Center for Maritime Logistics and Services CML, Am Schwarzenberg-Campus 4, 21073 Hamburg, Germany; 2 Present Address: Hapag-Lloyd AG, Ballindamm 25, 20095 Hamburg, Germany

**Keywords:** Maritime logistics forecasts, Empty container relocation, Machine learning, Mixture density network

## Abstract

Due to imbalances in the global transport of containerised goods, liner shipping companies go to great lengths to match the regional supply and demand for empty containers by transporting equipment from surplus to deficit regions. Making accurate forecasts of regional empty container availability could support liner companies and other involved actors by making better relocation decisions, thus avoiding unnecessary transport costs of empty equipment. Previously proposed container availability prediction models are limited to the application in individual regions and typically characterized by a high degree of temporal aggregation. Against this background, this paper introduces two novel approaches based on machine learning and probabilistic techniques to predict the future weekly availability of empty containers for more than 280 locations worldwide. The machine learning and probabilistic prediction models are built by analysing a unique data set of more than 100 million events from past container journeys. These events represent different stages during the transport process of a container. Both models use a two-step forecast logic. First, the expected future location of a container is predicted. Second, the expected timestamp for arriving at that location is estimated. The machine learning model uses artificial neural networks and mixture density networks to forecast the movements of containers. The models are quantitatively assessed and compared to the actual availability of containers and two more conventional forecasting approaches. The results indicate that the probabilistic prediction approach can keep up with conventional approaches while the neural network approach significantly outperforms the other approaches concerning every evaluation metric.

## Introduction

### Background

Freight transport within supply chains and to consumers is a critical component of a globalised world. The timely availability of raw materials and finished goods plays an essential role for most companies. Accordingly, the efficient movement of cargo supports production, trade, and consumption (Crainic 2003: pp.1–3). Transport companies depend on the availability of empty containers with suitable characteristics in terms of equipment size, type, and condition to fulfil any transport order. In the ideal case–the balanced trade–containers would be stripped and filled with new cargo at the same location shortly after. Where this is impossible, empty equipment must either be stored in a depot until its subsequent use or transported to a region with an empty container deficit. Such relocations of containers from import-dominant to export-dominant areas take place locally (e.g. from one terminal to another), regionally (e.g. from one location to another) or globally (e.g. from one country to another) (Theofanis and Boile 2009; Di Francesco et al. 2009). High costs are associated with container repositioning, primarily due to over-land and marine transport and storage and handling of containers (Karmelić et al. 2012; Moon et al. 2013).

Empty containers make up a significant part of the container transports—up to 40% in over-land and 20% of marine transports (Konings and Thijs 2001; Karmelić et al. 2012: p.223). It is estimated that costs of empty container relocations amount to $15 billion to $20 billion annually, representing around 5% to 8% of the carriers’ total costs (Sanders et al. 2015). Hence, liner shipping companies go to great lengths to match the regional supply and demand for empty containers by optimising equipment repositioning to minimise costs. Additionally, the carrier reduces the risk of a competitor receiving an order and can realise the transports himself by satisfying the empty container demand at each port (Crainic 2003).

Liner shipping companies have various options to reduce the costs related to empty container logistics. Most common, lower transport costs in import-dominant regions, sharing equipment between companies, or collaborating with competitors are used in practice. Another opportunity arises from recent advances in data-driven and machine learning approaches. Due to transport processes digitalisation in the maritime industry 4.0, an increasing amount of transport-related data is collected daily. This enables the application of data-driven decision support models in various use cases, e.g. stowage planning (Shen et al. 2017), fuel management (Fagerholt et al. 2015), and container inspection (Klöver et al. 2020). In other logistic domains, such models, especially using machine learning techniques, are already applied to support business decisions, such as guiding self-driving vehicles (van Meldert and Boeck 2016; Jeon et al. 2011) or optimise warehouse picking operations (Seward 2015). Where predictive models succeed in estimating supply and demand for empty containers, they possibly enable shipping companies to:

- Realise container relocations in time if a surplus or deficit is foreseeable,
- Adjust transport prices to meet expected market developments (dynamic pricing),
- Plan transport processes by considering future container availability to minimise transport costs.

Overall, an accurate estimation of empty containers’ future demand and supply contributes to container fleets’ efficient utilisation. Moreover, reducing regional and interregional equipment repositioning can also lower transport emissions (Crainic 2003; Schlingmeier 2017).

### Related literature

Several scientific papers still cover data-driven approaches to forecast container transport and port handling volumes. This also includes machine learning-based prediction models. For example, Shankar et al. (2020) use long-short-term-memory neural networks (LSTMs) to forecast container handling at the port of Singapore based on the historic quarterly throughput from 1995 to 2018 and compare the performance of their model to various time-series forecasting approaches. Tsai and Huang (2017) use an artificial neural network approach to predict container movements between ten Asian ports. They train a regression model using features extracted from more than one million port-to-port container transports recorded in Taiwanese customs clearance documents. Additionally, they use financial data, like the gross domestic product, interest rates and the industrial production index, as features.

Another example is found in Xiao et al. (2012), who use a hybrid model which combines autoregressive integrated moving average (ARIMA) (cf. Box et al. 2015) with artificial neural networks to forecast univariate container throughput data from Tianjin Port. Chan et al. (2019) compare various data-driven models to forecast container throughput of the port of Ningbo-Zhoushan; their data basis is the yearly handling volumes between 2004 and 2015. Starting from a similar database of quarterly container throughput in North Adriatic ports, Dragan et al. (2014) test different data-driven approaches, including ARIMA, to forecast regional container throughput on a port level.

### Research design

Even though several authors proposed and tested methods to forecast container throughput at ports, most are characterised by:

- Relatively small data volume, which prevents training robust models,
- High degree of temporal aggregation (e.g. yearly or quarterly throughput),
- No differentiation of container types, and
- Predictions that cover only small regions or even individual ports.

This paper tries to overcome these shortages. Two novel data-driven models are introduced–a deep learning model and a probabilistic model–to estimate the future availability of empty containers of different types for more than 280 locations worldwide. The machine learning model consists of feed-forward neural networks (cf. Bishop 2006) and mixture density networks (Bishop 1994). The prediction models are built by analysing a unique data set of more than 100 million events from past container movements. The data set is part of the research project C-TIMING (ConTainer Availability Index Made In Germany),^1^ which is funded by the Federal Ministry of Education and Research of Germany. The data was collected by the project partner xChange Solutions (xChange),^2^ an online platform for leasing and trading containers. The transport information was gathered from liner shipping companies by using Application Programming Interfaces (APIs) and tracking-crawlers. The events in the data set represent different states of the transport process of a container, in particular, these are: ‘dispatch at container depot’, ‘loading on ship’, ‘discharge from ship’ and ‘return to container depot’.

While related work mainly proposes forecasting the port throughput based on historical economic data of the ports (e.g. Dragan et al. 2014; Pang and Gebka 2017; Shankar et al. 2020), the models presented in this paper are built on journey data of individual containers. The subsequent events of each container are predicted for a large set of containers by forecasting the expected event location and realisation time. Based on the individual forecasts for a high number of containers, the supply and demand for various types of empty containers at worldwide locations are estimated. The proposed approach uses heterogeneous features for the prediction and enables the estimation of container availability for any port in the world.

The remainder of the paper is structured as follows: First, the data set used to train and evaluate the prediction models is introduced in Sect. Data set. Subsequently, Sect. General prediction approaches briefly describes the general prediction approach and introduces the two prediction models. Next, the performance of the models is evaluated in Sect. Results. Section Discussion of results discusses the results and practical and academic implications are provided. Finally, Sect. Conclusions summarises this article.

## Data set

### Events in the data set

**Fig. 1:**
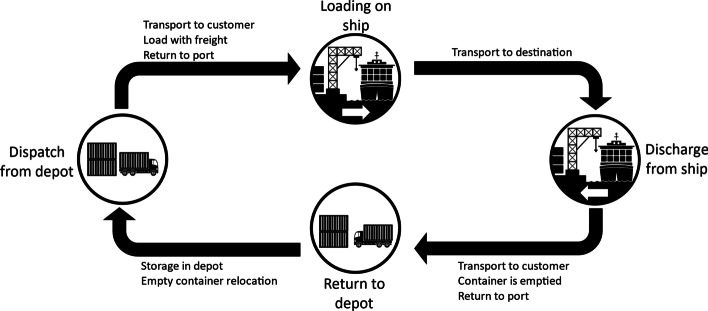
Events during the transport process of containers. *Source*: Own figure

The machine learning and statistical prediction models are built by analysing a unique data set of more than 102 million events from past container journeys. These events represent different states during the transport process of a container, in particular the events ‘dispatch at container depot’ (event name: dispatch), ‘loading on ship’ (loading), ‘discharge from ship’ (discharge) and ‘return to container depot’ (return). Between these four events, different actions might take place. Usually, the container is taken out of a depot, transported to the customer, filled by the customer, and returned to the port between a dispatch at the container depot and the subsequent loading on the ship. After loading, the container is transported from the origin to the destination before it is discharged from the ship. Subsequently, the container is transported to the customer, emptied and returned to the depot’s location. The container is stored in the depot between a return and a subsequent dispatch from the container depot. Eventually, an empty transport to another location can occur between these two events. However, every action between the four main event types is not captured in the data set. Figure 1 depicts the events of a container journey.

**Fig. 2:**
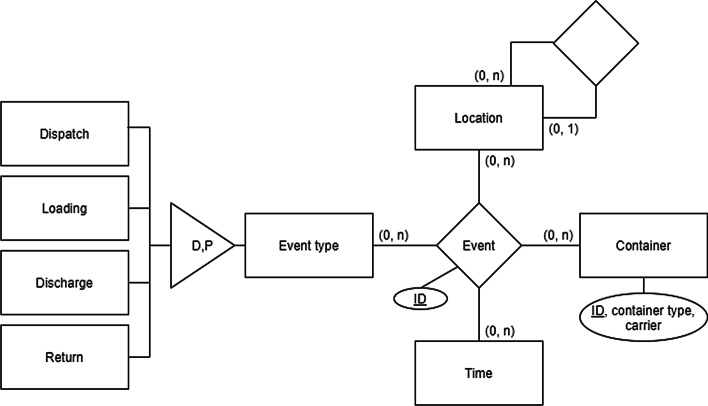
Entity-relationship model of events. *Source*: Own figure

Each event in the data set can be identified by a unique ID and belongs to a specific container. The type of the event (dispatch, loading, discharge, return) is provided and the location and time stamp. The events of the data set represent the movements of laden containers solely. The transport of empty containers is not included in the data set. Therefore, a container ID can identify the containers in the data. Additionally, the type (e.g. forty-foot high cube) and the owner (carrier) of the container is provided. Figure 2 visualises the information related to an event with an entity-relationship model (cf. Chen 1976).

### Descriptive analysis of the data

**Fig. 3:**
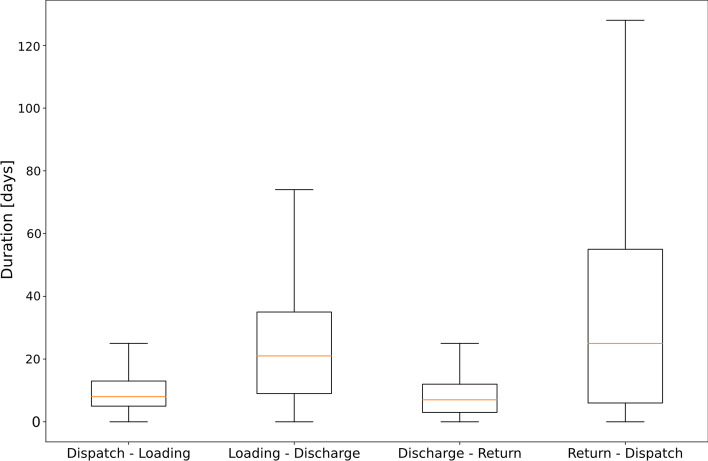
Duration between subsequent events. *Source*: Own figure

Insights of a descriptive analysis of the data set are provided in the following section. The average time between two subsequent events of a container in the data set is 19.7 days. However, the duration between subsequent events differs for each event type. For example, while the average time between a loading and a subsequent discharge (*transport time*) corresponds to 23.5 days (lower quartile: 9 days, upper quartile: 34 days), the time between a return and a dispatch (*dwell time*) averages to 35.2 days (lower quartile: 5 days, upper quartile: 55 days). Figure 3 depicts the duration distribution between subsequent events with box and whiskers plots (cf. Tukey 1977).

The data’s transport events occurred at more than 100,000 locations worldwide. These locations include most of the ports and container terminals in the world. However, several locations’ data quantity was insufficient to train prediction models robustly. Transport events’ geographic information was preprocessed to mitigate this issue; the locations were mapped either to a port or a country. Therefore, based on their domain knowledge, 83 ports were selected by xChange’s domain experts representing the world’s most important ports like Shanghai, Ningbo, and Rotterdam. The remaining locations were mapped to their corresponding country. A total of 204 countries are included in the data set.

**Fig. 4:**
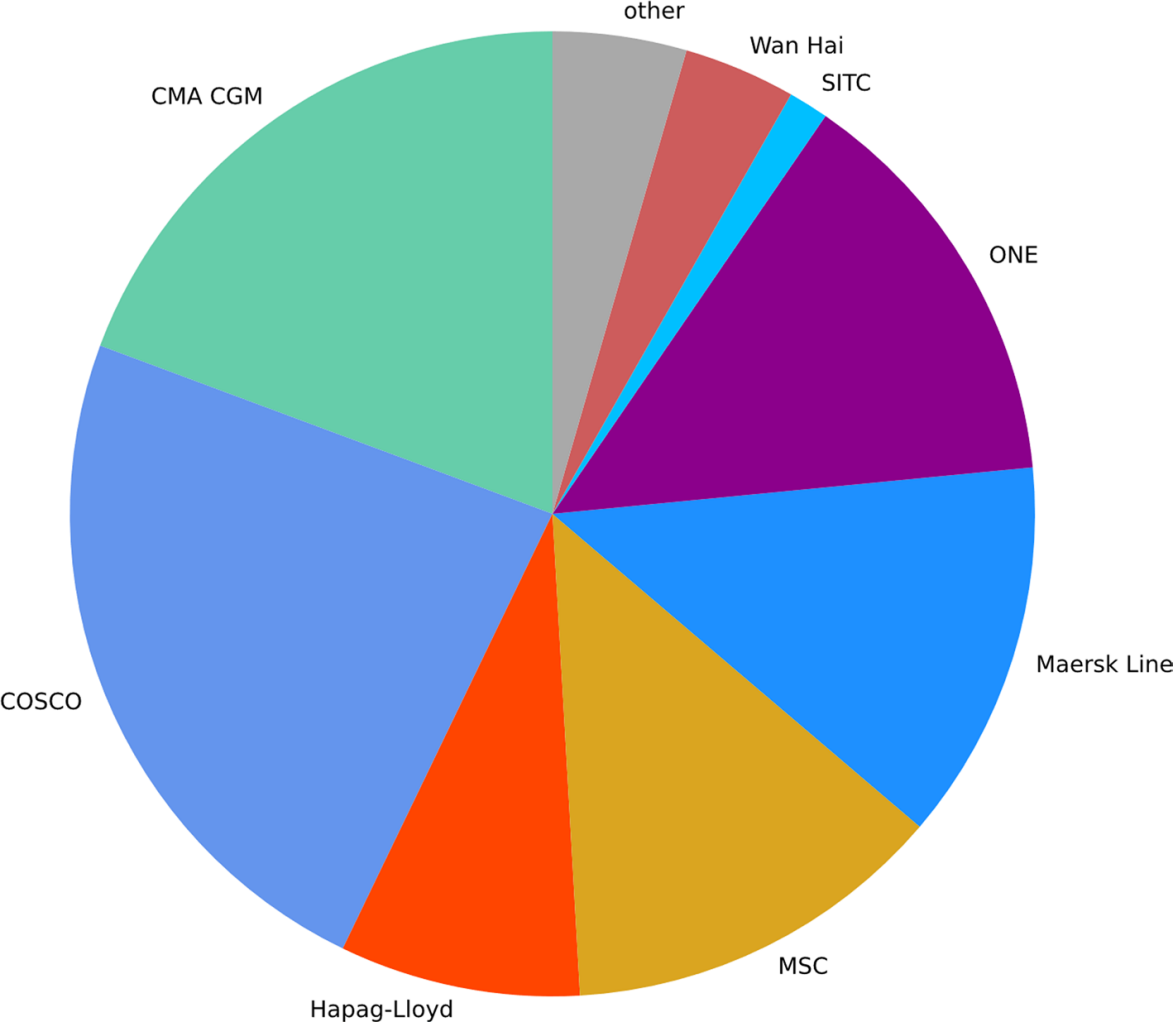
Share of liner shipping companies by number of movements. *Source*: Own figure

The containers part of the data set belong to one of 60 liner shipping companies, including container movements of each of the 14 largest liner shipping companies.^3^ However, the share of the events of a carrier does not correspond to its market share: For example, even though COSCO’s market share corresponds to 12.2% as of July 2021,^4^ 24.4% of the events relate to a COSCO container while just 12.9% of the events are of containers of the largest market player Maersk. Figure 4 visualises the share of the events of the liner shipping companies in the data.

Containers of 23 different types are included in the data. These container types include the most common variations of containers (e.g. dry container, reefer, flat rack, open top) with a length of either twenty-, forty- or forty-five-foot. According to Che et al. (2011: p. 506), twenty-foot, forty-foot dry and forty-foot high cube containers are among the most commonly used container types. These three container types account for 92.5% of the events in the data.

**Fig. 5:**
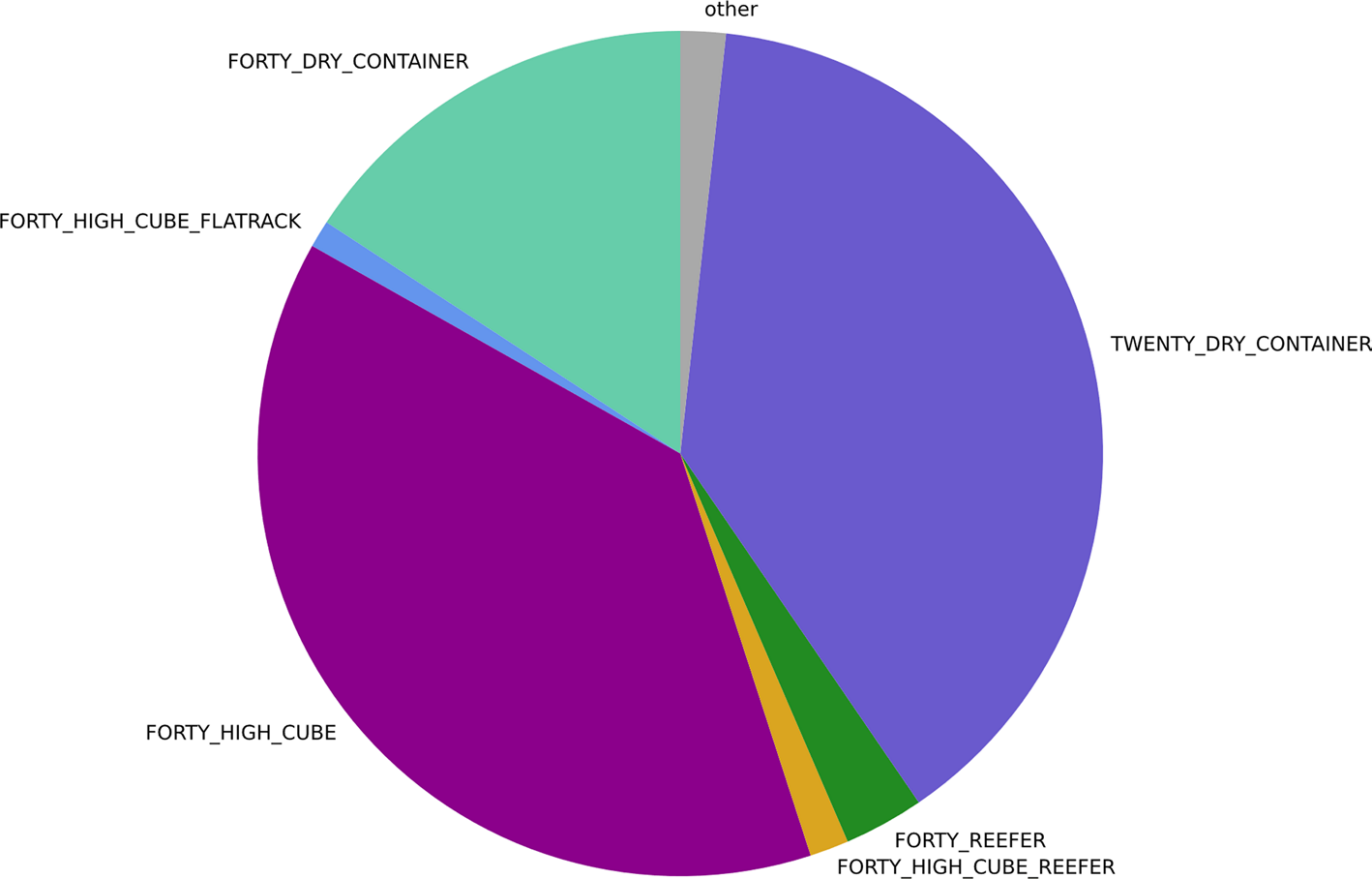
Share of container types. *Source*: Own figure

Figure 5 visualises the share of the events of the different container types.

**Fig. 6:**
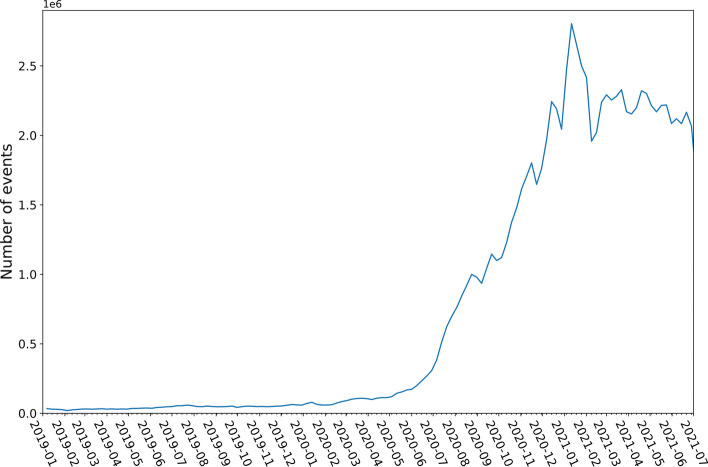
Weekly number of events in the data set. *Source*: Own figure

The events in the data set are collected over more than 30 months: The first events date back to 1st January 2019 and the last to the 22nd July of 2021. 102,214,134 events of 12,893,347 containers are included in the data. Even though it covers a long period and contains a large number of total events, several challenges are associated with the available data set. The most important challenge is that the weekly events increase considerably over the available period. While 219,653 events are tracked over the first two months of the covered period, 19,108,155 events are tracked in May and June 2021, cf. Fig. 6. Hence, most of the containers in the data are not tracked over the whole period but only for some sporadic events over some weeks or months.

Furthermore, there are both missing events (or completely missing journeys) and inconsistencies in the data on a limited but not negligible level, even though the quality and completeness of the data increase continuously over the period covered by the data set. All these aspects represent challenges to extracting information on the future demand and supply of empty containers and thus influence the quality of the models trained with the data.

## General prediction approaches

To support the determination of the regional empty container availability, the forecasting approaches aim to estimate the number of containers returned to the container depot and dispatched at the container depot at any location in the data set. The approach to estimating the number of returns and dispatches at the container depots presented in this paper is to forecast individual containers’ movements (events). For each movement of a container, the location and date of the subsequent events has to be estimated. Then, based on the forecasts of the individual containers, the total number of returns and dispatches at the regional container depots can be derived, e.g. by a database query. The novelty of this prediction approach is that the future container throughput is forecasted by considering the current state of the global container fleet. Thereby, the model should anticipate the effects of the current situation. For example, suppose that a significant container shortage in an export-dominant region is observed at a certain point in time. In that case, the model can anticipate that this shortage causes the import to decline at the port, which mainly receives the freight from this region.

The prediction approach of a single container movement consists of five steps: First, features representing descriptive characteristics are derived from the last event, e.g. container type, location, or the time passed. Subsequently, a destination prediction model processes these features, producing a probability for each considered location to be the destination. Based on this probability distribution, one location is sampled as the destination. Next, the defining features and the predicted destination are passed to a duration prediction model, which outputs another probability distribution representing the transition times of the container from the origin to the predicted destination. Again, one duration is sampled from the probability distribution. Finally, the date of the next container event is determined by adding the sampled duration to the date of the last event of the container. Subsequently, multiple upcoming container events can be estimated by repeating the prediction process with already forecasted events. This paper introduces two different prediction models, which implement the described prediction approach. The first model uses two artificial neural networks to forecast the locations and dates of future events, while the second is a probabilistic model.

### Neural network approach

The first approach combines two different types of neural networks to forecast the movements of an individual container as described by the general prediction approach. First, a feed-forward neural network (Bishop 2006, cf. Chapter 5 ff.) is used to predict the next event’s location. Subsequently, a mixture density network (cf. Bishop 1994) is utilised to estimate the transition time based on the descriptive features of the last event and the predicted location. An extensive grid search determined the hyperparameters of the neural networks presented in the following. Information about the grid search is provided at the end of this section.

#### Destination prediction

To generate a probability distribution representing the probability ${p}_{l}$ of each location ${l}_{i}\in L$ to be the location of the next container event, where $L$ corresponds to the set of all locations in the data set, a feed-forward neural network is used. The models’ inputs are descriptive features representing selected characteristics of the last event and the associated container. Table 1 briefly describes the features used to train the destination prediction model. The column *type* indicates whether the feature is categorical or numeric. A categorical feature can only take on a limited number of values. E.g. for the categorical feature event type, the set of possible values consists of the four event types dispatch, loading, discharge, and return. The column *unique values* shows the amount of unique values for each categorical feature based on their presence in the data set. Numeric values are not restricted to a limited number of values.

**Table 1:** Features of the destination prediction model. *Source*: Own elaboration

Feature	Description	Type	Unique values
Origin	Location of the last event of the container. This can either be a specific port or the country were the event occurred	Categorical	287
Container type	String-based indicator of the type of the container, e.g. *FORTY-DRY-CONTAINER*	Categorical	23
Carrier	String-based indicator of the carrier of the container, e.g. *MSC*	Categorical	60
Days passed	Number of days passed between the last event of the container and the date the forecast is made	Numeric	N.A
Event type	String-based indicator of the type of the last event, either dispatch, loading, discharge, or return	Categorical	4

An individual feed-forward neural network is trained for each of the four event types in the presented approach. Even though it is expected that the forecasting approach can be successfully applied if the event type is included as a feature; hence, only one destination prediction model needs to be trained. Each categorical feature is passed as a one-hot encoded vector to the model.

The destination prediction models are utilised to output a probability distribution given the descriptive features of an event as input. Each probability distribution is supposed to assign a probability ${p}_{{l}_{i}}$, with $0\le {p}_{{l}_{i}}\le 1$ for each location ${l}_{i}\in L$, in such a way that $\sum_{i}^{|L|}{p}_{{l}_{i}}=1$. The output of the neural network is scaled with the softmax activation function (cf. Nwankpa et al. 2018) to satisfy this constraint.

The features are processed by three fully-connected layers consisting of 750 neurons with Rectified Linear Unit (ReLu) (cf. Nair and Hinton 2010) as activation function before they are passed to the output layer. The models are trained with the historic events of the data set. Thereby, the single location of the next event serves as target. The proposed model can learn to assign a probability for each location even though only a single destination is passed as target by learning from multiple events described by the same or similar features. The destination prediction models are trained for 10 epochs with the adam optimiser with a batch size of 1028.

#### Duration prediction

Mixture density networks are used to estimate the date of the next event. Mixture density networks are a special type of neural network designed to output a mixture model given some input features (cf. Bishop 1994). A mixture model can be thought of as a probabilistic model consisting of $k$ components ${C}_{1},\dots , {C}_{k}$ (or subpopulations) which describe the characteristics of a collection of elements (population). Each component itself is a probabilistic model (e.g. probability density function) representing the characteristics of a subgroup of the overall population (Baxter 2010).

**Fig. 7:**
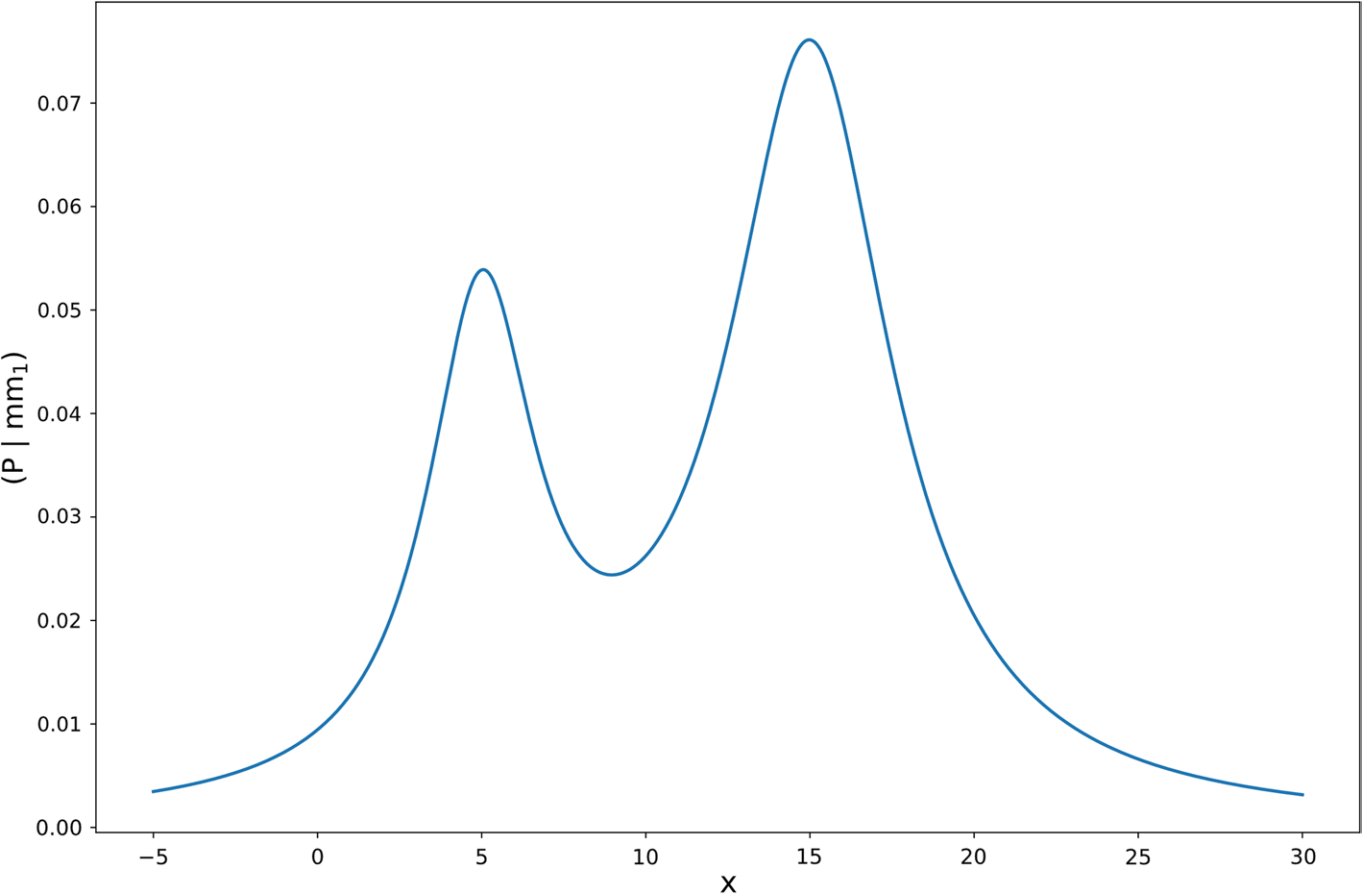
Exemplary mixture model. *Source*: Own figure

A component can be parametrised by different values depending on the type of probability distribution of the component (e.g. Gaussian distribution, exponential distribution). For example, if the component ${C}_{1}$ is a Gaussian distribution, the component can be represented by two values: mean (${\mu }_{1}$) and standard deviation (${\sigma }_{1}$), hence ${C}_{1}=\mathcal{N}({\mu }_{1}$, ${\sigma }_{1})$. Additionally, each component of a mixture model is weighted by a weighting factor ${\alpha }_{1},\dots , {\alpha }_{k}$, with $\sum_{i}^{k}{\alpha }_{i}=1$. Figure 7 visualises a mixture model consisting of two components, ${mm}_{1}=P\left(x|{C}_{1}, {C}_{2},{\alpha }_{1},{\alpha }_{2}\right).$ The first component is a Gaussian distribution with a mean of $5$ and a standard deviation of $2$ (${C}_{1}=\mathcal{N}(5, 2)$) and the second component a Gaussian distribution with a mean 15 and standard deviation of 3 (${C}_{2}=\mathcal{N}(15, 3)$). The first component is weighted by ${\alpha }_{1}=0.3$ and the second component by ${\alpha }_{2}=0.7$ (cf. Baxter 2010).

Mixture density networks estimate the parameters of a mixture model. Hence, the mixture density network needs to output for each component a weighting factor $\alpha$ and the parameters of the distribution, e.g. $\mu$ and $\sigma$. In case all components can be parametrised by ${\mu }_{1}, \dots , {\mu }_{k}$ and ${\sigma }_{1}, \dots ,{\sigma }_{k}$ the output $o$ of the mixture density network can be thought of as a vector $o=\left\{\mathrm{\rm M},\Sigma , \mathrm{\rm A}\right\}$, where $\mathrm{\rm M}=\left[{\mu }_{1}, \dots , {\mu }_{k}\right]$, $\Sigma =\left[{\sigma }_{1}, \dots ,{\sigma }_{k}\right]$ and $\left[\mathrm{\rm A}={\alpha }_{1},\dots , {\alpha }_{k}\right]$. To satisfy the constraint that $\sum_{i}^{k}{\alpha }_{i}=1$, softmax is applied to Α. A slightly modified exponential linear unit (ELU) activation (cf. Clevert et al. 2015) is applied to the remaining output values of the model (${\uplambda }_{1}\left(x\right), {\uplambda }_{2}\left(x\right))$ to prevent that the values of $\mathrm{\rm M}$ and $\Sigma$ become smaller or equal to zero (cf. Borchers 2019):

$${\text{M}}\left( x \right) = {\text{ELU}}\left( {\lambda_{1} \left( x \right)} \right) + 1,\;{\text{respectively}}$$

$${\Sigma }\left( {\text{x}} \right) = {\text{ELU}}\left( {{\uplambda }_{2} \left( x \right)} \right) + 1.$$

The model learns the values of $\mathrm{\rm M},\Sigma ,$ and $\mathrm{\rm A}$ by minimising the average negative log-likelihood of the ground truth values (transition times of the containers between the events) to be represented by the mixture model constructed by the mixture density network (cf. Borchers 2019). Thereby, the model is supposed to learn to construct the mixture model to reflect the distributions of the historical data.

The presented models are composed of three fully-connected layers with 450 neurons. The input to the model is a vector representing the one-hot encoded origin, destination, carrier, and container type. Thereby, the model is supposed to learn to output a mixture model for each input feature vector to reflect the distributions of the historical data. Our best-performing mixture density networks are combined from two Cauchy distributions and one Gaussian distribution. One mixture density network is trained for each event type as for the destination prediction. The models are trained for 30 epochs with the adam optimiser and a batch size of 1024. During the inspection of the model’s output, it was observed that the model could learn realistic distributions even for feature combinations that are not part of the historical data by generalising from historical data of similar feature combinations.

**Fig. 8:**
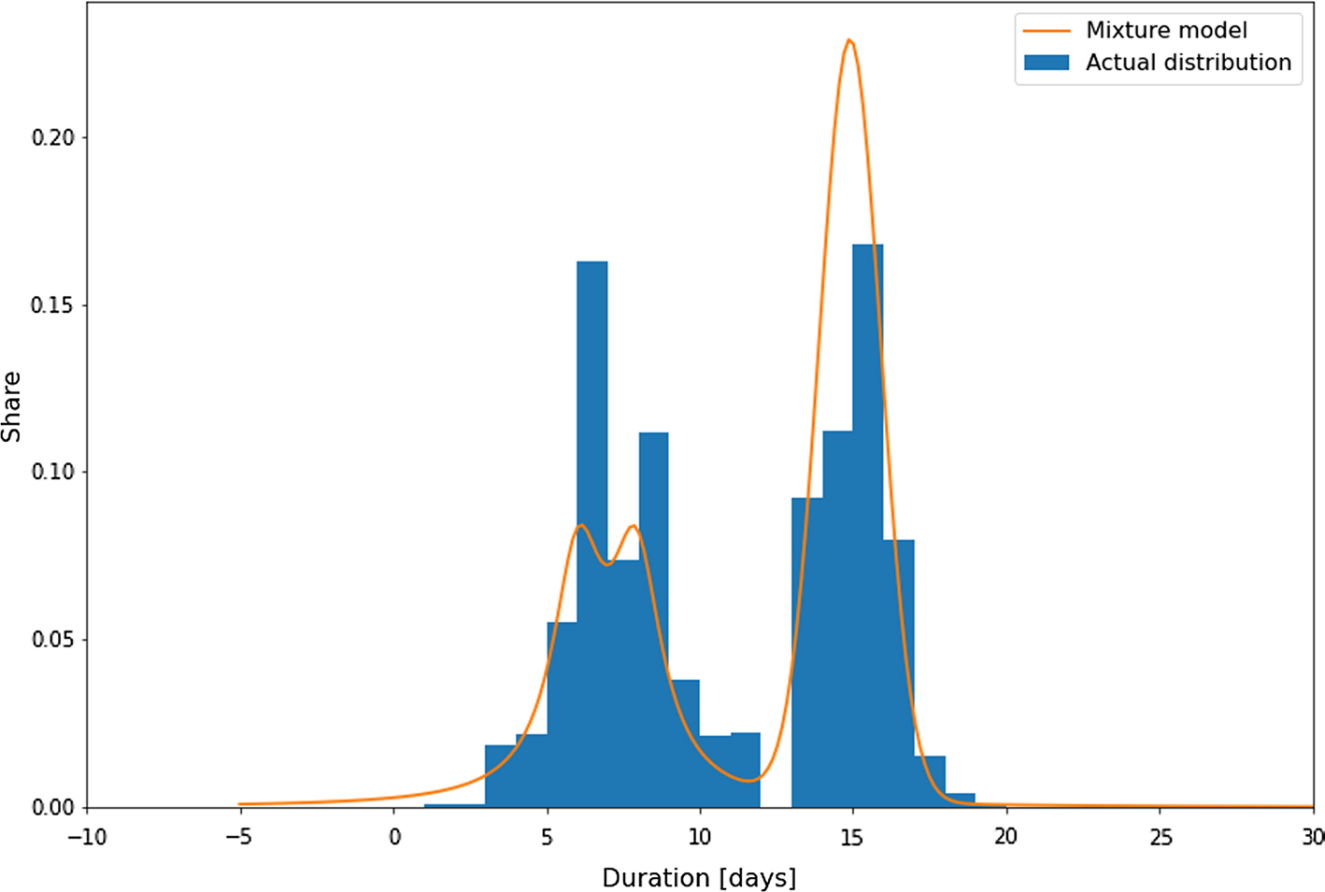
Predicted mixture model for transport time from Shanghai to the United States. *Source*: Own figure

Figure 8 depicts the mixture model for the transport time learnt by the mixture density network and the distribution of the actual values of the following features: the port of Shanghai is selected as the origin, the United States as the destination, Wan Hai as the carrier, and no specific container type is provided. Three thousand five hundred eighty-nine events with this feature combination are within the training data.

To determine the date of the next event of a container, a duration ${d}_{sel}$ is sampled from the mixture model provided by the mixture density network—given the descriptive features of the last event of the container—and added to the date of the last event. The selected duration ${d}_{sel}$ has to satisfy the constraint ${d}_{sel}>{\delta }_{event}$, where ${\delta }_{event}$ corresponds to the days that have passed since the last event. Thereby, it is ensured that the next event will happen.

#### Hyperparameter optimization

The presented hyperparameters of the destination and location prediction model were proposed by a grid search (cf. Montgomery 2020). The grid search was organized in multiple iterations. In the first iteration, a sparsely populated set of hyperparameter values was definded for each hyperparameter listed in Table 2. For example, the initial hyperparameter values of the hyperparameter *number of layers* included 1, 3, 9, 27, 54, and 81 layers. Next, a set of initial hyperparameter values was specified for each hyperparameter similarly. The hyperparameter search space was defined by the cartesian product of hyperparameter values of each hyperparameter. Finally, one model was trained for each hyperparameter combination of the hyperparameter space.

**Table 2:** Hyperparameters of the best-performing neural networks. *Source*: Authors

Hyperparameter	Destination prediction modell	Duration prediction modell
Number of layers	3	3
Number of neurons	750	450
Epochs	10	30
Batch size	1028	1024
Activation function	ReLu	ReLu
Activation function (last layer)	Softmax	Softmax and NNelu
Loss function	Categorical cross-entropy	Negative log-likelihood
Optimizer	Adam	Adam
Initialization	Xavier	Xavier
Mixture model	–	3 distributions (2 × moyal distribution, 1 × normal distribution)

Hyperparameters that negatively influenced the models’ metrics were excluded from the hyperparameter space for the following iterations while additional hyperparameters with similar values to the best performing values were added. For example, the values 1, 27, 54, and 81 were discarded, and the values 2, 4, 6, 8, and 12 were included for the second iteration for the hyperparameter number of layers. The described process was simultaneously carried out for all the hyperparameters listed in Table 2. Table 2 shows the hyperparameter combination for the destination and duration prediction model yielding the best performance.

### Probabilistic model

The probabilistic model follows the same prediction procedure as the neural network approach. However, the learning process is simpler than the neural networks. The general idea is that the predicted location and duration is the same as one historical event described by the same or similar descriptive features. Thus, the probabilistic model used three features (origin, event type and days passed) to predict the location and four features (origin, destination, event type and days passed) to estimate the date of the next event.

The probabilistic model learns a probability distribution for each feature combination from the historic container movements. Thereby, the feature combination serves as input for the probabilistic model. The probabilistic model outputs a location duration based on the input feature combination. The probability distribution assigns a probability ${p}_{{l}_{i}}$, with $0\le {p}_{{l}_{i}}\le 1$ for each location ${l}_{i}\in L$, in such a way that $\sum_{i}^{|L|}{p}_{{l}_{i}}=1$. The probability distribution for each feature combination depends on the historic destinations and respectively duration of containers with feasible feature combinations. A feature combination is considered feasible if the location-based features and the event type are the same as the descriptive features of the last event of the container and the duration is larger than the days passed since the last event of the container under consideration.

Following the historical data, consider the following example in which 10 containers were shipped from Lisbon. Three of those were discharged from the ship 50 days later in Shanghai, and two after 70 days in Shanghai. Three arrived in New York after 28 days. The remaining two were sent to Hamburg, Germany, and discharged after $17$ days. If the probabilistic model is used to predict the next location and duration of a container that was loaded in Lisbon $20$ days ago, then the probability that Shanghai is selected as a destination corresponds to $\frac{5 }{8} = 62.5\%$. In case Shanghai is sampled as the destination, the statistical model selects a shipment duration of $50$ days with a probability of $\frac{3}{5} = 60\%$.

### Evaluation approach

The objective of the forecasts is to estimate a weekly container availability index of specific container types (either twenty-foot or forty-foot) at each location in the data set. The container availability index (CAX) is calculated following the approach used by xChange,^5^ which is defined as the number of containers of a given type $ct$ (e.g. twenty-foot standard container) returned at a specific location $l$ during a certain week $w$ (${returns}_{l, ct,w}$) divided by the sum of returns ${returns}_{l, ct,w}$ and the number of dispatches of containers with the same type $ct$ at location $l$ within week $w$ (${dispatches}_{l, ct,w}$).

$${CAX}_{l,ct,w}=\frac{({returns}_{l, ct,w}) }{\left({returns}_{l, ct,w}\right) + ({dispatches}_{l, ct,w})}$$

The absolute error and squared error between the predicted and actual container availability for each evaluation feature combination (combination of location $l$, week $w$ and container type $ct$) in the evaluation set is calculated to quantify the quality of the container availability index predicted by the models. The mean absolute error (MAE) respectively mean squared error (MSE) is computed by averaging the absolute errors respectively squared errors over all evaluation feature combinations of the evaluation set. A small MAE and MSE indicate that the predicted container availability indices are similar to the actual data values in the evaluation set. If the MAE of two different models are quite similar, but the MSE of one model is significantly larger than the MSE of the other, it indicates that the model with the higher MSE predicts for at least one evaluation feature combination a significantly different container availability index than reflected by the actual data.

The models are evaluated on rolling forecasting origins (cf. Hyndman and Athanasopoulos 2018): multiple evaluation data sets representing different time spans are used to quantify the performance of the two prediction models. The evaluation data is selected following the sample-out approach for each forecasting origin: events that took place before the forecasting origin are used for training the models, while the objective of the models is to predict the event after the forecasting origin. Each evaluation data set covers the events of a time span of 12 weeks. Containers in the evaluation data set have 4 to 5 transport events on average during 12 weeks with a maximum of 20 events. The prediction models are trained and evaluated on 18 forecasting origins. The first forecasting origin is 01.11.2020 and the last 28.02.2021.^6^ The training data includes all the events dating back to January 2019.

In addition to the actual values, the quality of the two prediction models is compared to two benchmark functions, Benchmark Naïve and Benchmark SES. Benchmark Naïve predicts the same number of events for each evaluation feature as in the last week before the forecasting origin. Benchmark SES instead applies a simple exponential smoothing on the amounts for each feature combination over the 12 weeks before the forecasting origin (cf. Hyndman and Athanasopoulos 2018, Chapter 7.1).

The number of events in the data set increases weekly, and the share of four event types is not evenly distributed due to missing data, as described in Sect. Data set. This leads to a bias regarding the number of events happening at the ports each week. Therefore, each time span’s evaluation data must be cleaned to mitigate this issue. The basic idea of the cleaning process is that containers are only included in the evaluation data, which are (probably) constantly tracked over a time span and thereby show realistic movements. Four criteria are defined that must be satisfied by a container to be included in the evaluation data set:

1. The container is tracked at least 150 days before the forecasting origin.
2. The events of the container happen in the expected order (dispatch $\to$ loading $\to$ discharge $\to$ return $\to$ dispatch).
3. The time between two consecutive container events is never larger than 150 days.
4. The container has at least one event during the 150 days after the last day of the evaluation time span.

Criterion 1 mitiages issues related to the data collection process: The amount of containers tracked increases weekly, and the tracking of a container typically begins if a container is dispatched from depot. This leads to the issue that the first events of the transport process are over-represented in the data set. By only considering containers already tracked for 150 days,^7^ this challenge can be significantly mitigated. Criteria 2–4 aim to ensure that the container is constantly tracked. Therefore, the events have to occur in the expected order (criterion 2). Furthermore, there should be no missing container transports (criterion 3) without excluding empty container transports–which are not included in the data set. The cut-off for this criterion was set to 150 days by domain experts because it is more likely that the container is no longer tracked than that it does not have a single transport event within this period. Criterion 4 ensures that the container is still tracked after the evaluation period. If criterion 4 is not applied, it was observed that there is an over-representation of return events in the evaluation data since most of the missing data issues originate from absent dispatch events after the return to the depot of a container.

## Results

This section presents the forecast results of our two prediction models over the 18 forecasting origins compared to the predictions of the benchmarking approaches, and some insights are highlighted. Table 3 shows the MAE and MSE averaged over the 18 forecasting origins for the two prediction approaches and the two benchmarks for locations where at least 50 containers are returned or dispatched from the depot.

**Table 3:** MAE and MSE over the evaluation period. Source: Authors

	Mean absolute error		Mean squared error	
Approach	Twenty-foot	Forty-foot	Twenty-foot	Forty-foot
Neural Network	0.0753	0.0711	0.0093	0.0086
Probabilistic Model	0.1048	0.0870	0.0170	0.0122
Benchmark Naive	0.1086	0.0925	0.0194	0.0147
Benchmark SES	0.1018	0.0864	0.0176	0.0131

The forecasts of the neural network approach significantly outperform the other three approaches with a MAE of $0.0753$ for the twenty-foot container (MSE: $0.0093$) and $0.0711$ for the forty-foot container (MSE: $0.0086$). While the prediction performance of the neural network is promising, the accuracy of the probabilistic approach is similar to the accuracy of the two benchmark models. Even though the observation that the probabilistic model performs similarly to the two benchmarks is still promising: it indicates that the applied prediction approach, which derives the container availability based on forecasts of individual containers, can compete with predictions at port level.

As shown in Table 3, the prediction quality of each model is better for forty-foot containers than twenty-foot containers. Larger training and evaluation data availability for forty-foot containers might explain this observation, cf. Fig. 5. Generally, it was noticed during evaluation that the performance of the neural network approach seems to be positively influenced by the higher availability of training and evaluation data.

**Fig. 9:**
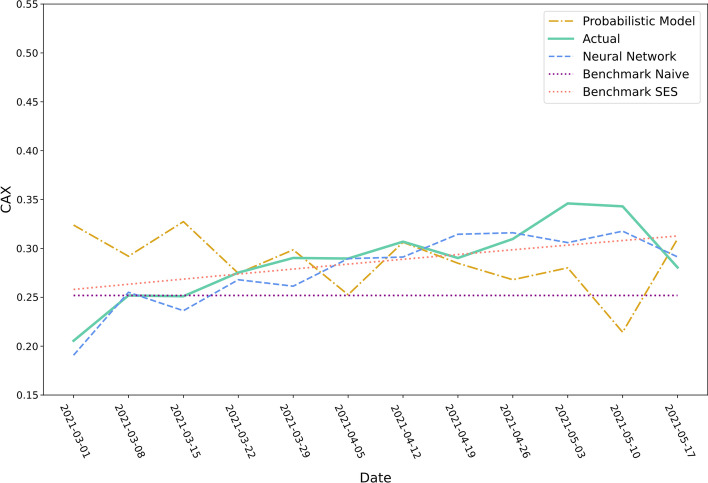
Predicted container availability for the port of Qingdao (forecasting origin: 01.03.2021). *Source*: Own figure

**Fig. 10:**
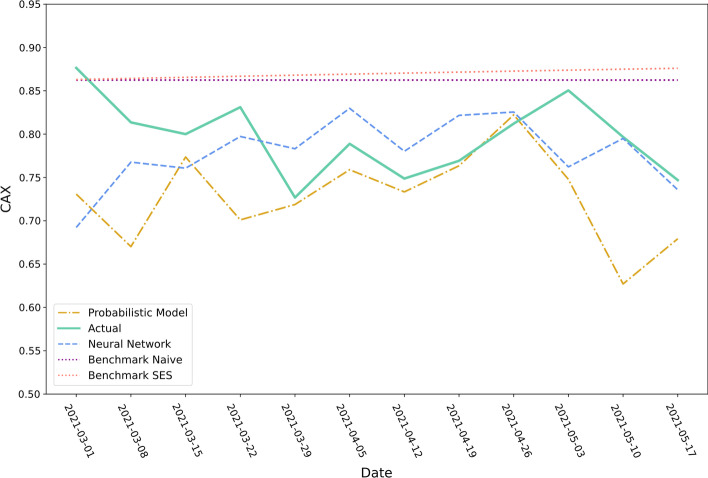
Predicted container availability for the port of Tokyo (forecasting origin: 01.03.2021). *Source*: Own figure

Figures 9 and 10 show the twelve-week container availability forecasts of the four approaches starting from the forecasting origin 1^st^ March 2021 for the ports of Qingdao and Tokyo. Over the depicted time period, the container availability index at the port of Qingdao increased from $0.21$ to $0.35$. The neural network approach and Benchmark SES accurately anticipate the increase while the other two models fail to forecast the observed pattern accurately. The observation is also captured by the MAE (cf. Table 4): The neural network approach and Benchmark SES have a MAE of 0.0160 respectively 0.0202 followed by Benchmark Naïve (0.0426) and the probabilistic model (0.0460).

**Table 4:** MAE of the prediction models for the port of Qingdao and Tokyo. *Source*: Authors

	Mean absolute error	
Approach	Qingdao	Tokyo
Neural Network	0.0160	0.0499
Probabilistic Model	0.0460	0.0712
Benchmark Naive	0.0426	0.0680
Benchmark SES	0.0202	0.0752
Total container	12,729	1,843

The container availability index development at the Tokyo port is more volatile and does not follow a strict pattern. All four approaches do not accurately forecast container availability in Tokyo. The predictions made by the neural network approach are the most accurate according to the MAE (0.0499), followed by the naïve benchmark (0.0680). The average weekly amount of containers in the evaluation data set handled at the port of Tokyo corresponds to 115 containers. In comparison, on average, 795 containers of the evaluation data set are dispatched or returned each week at the port of Qingdao. Hence, the volatility of the container availability index at the port of Tokyo might originate from the relatively small number of events at this port.

**Fig. 11:**
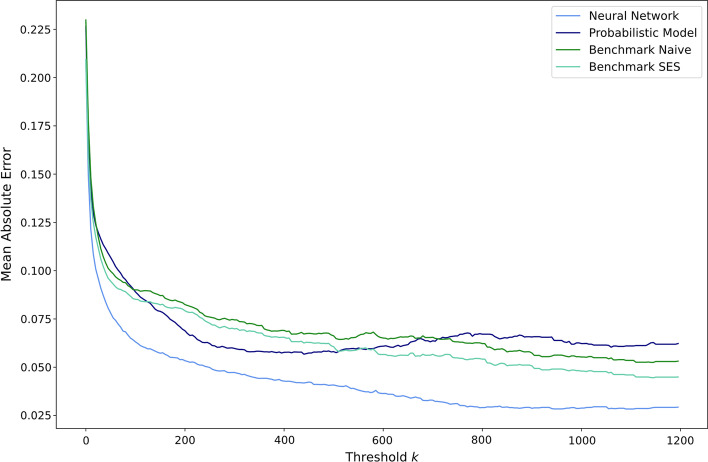
MAE for different subsets of the evaluation data set. *Source*: Own figure

To test the hypotheses that the forecast quality increases with a higher number of container events at the locations, the MAE was computed for different ${subsets}_{k}$ of the evaluation data set. In each ${subsets}_{k}$ locations with less than $k$ weekly events were excluded from the evaluation data set. For example, ${subsets}_{50}$ only includes the ports and countries where at least 50 container events occur weekly. Figure 11 depicts the MAE of subsets of the evaluation data set with different thresholds $k$, in such a way that it holds that $0\le k\le 1200$.

It can be seen in Fig. 11 that the predictive performance of the neural network approach, Benchmark Naïve and Benchmark SES–expressed by the MAE–nearly constantly increases with a higher number of container events at the locations of the evaluation data set. Only the probabilistic model does not follow this pattern. Instead, the prediction quality of the probabilistic model slightly decreases if $k$ is greater than $400$. Additionally, it can be observed that the MAE of all four approaches is relatively high if all locations are included in the evaluation set, indicating that the models do not accurately predict the container availability for locations with only a few weekly container events. This can mainly be attributed to the volatility of the container availability index at these locations. However, it can be seen that the predictive performance significantly increases if low-volume locations are excluded from the evaluation data set. At locations with more than $200$ weekly container events, the neural network approach achieves an MAE of approximately $0.05$. The metric even improves to $0.03$ if only locations with more than $800$ weekly events are included.

Furthermore, it can be seen that the model using neural networks outperforms the other three approaches for each threshold $k$. This observation also indicates that forecasts made on the individual container level can outperform predictions on the port level. The advantage of the neural network approach compared to predictions on the port level especially is that the presented approach can forecast the movements of the containers given the current state of the global container fleet. For example, if only a relatively small number of containers were loaded on vessels in Chinese ports over the last weeks, the presented prediction approach can anticipate that this situation will decrease discharged containers at ports that mainly import goods from China. Furthermore, the prediction approach can estimate the implications of this situation over the following weeks at every considered port and country.

**Fig. 12:**
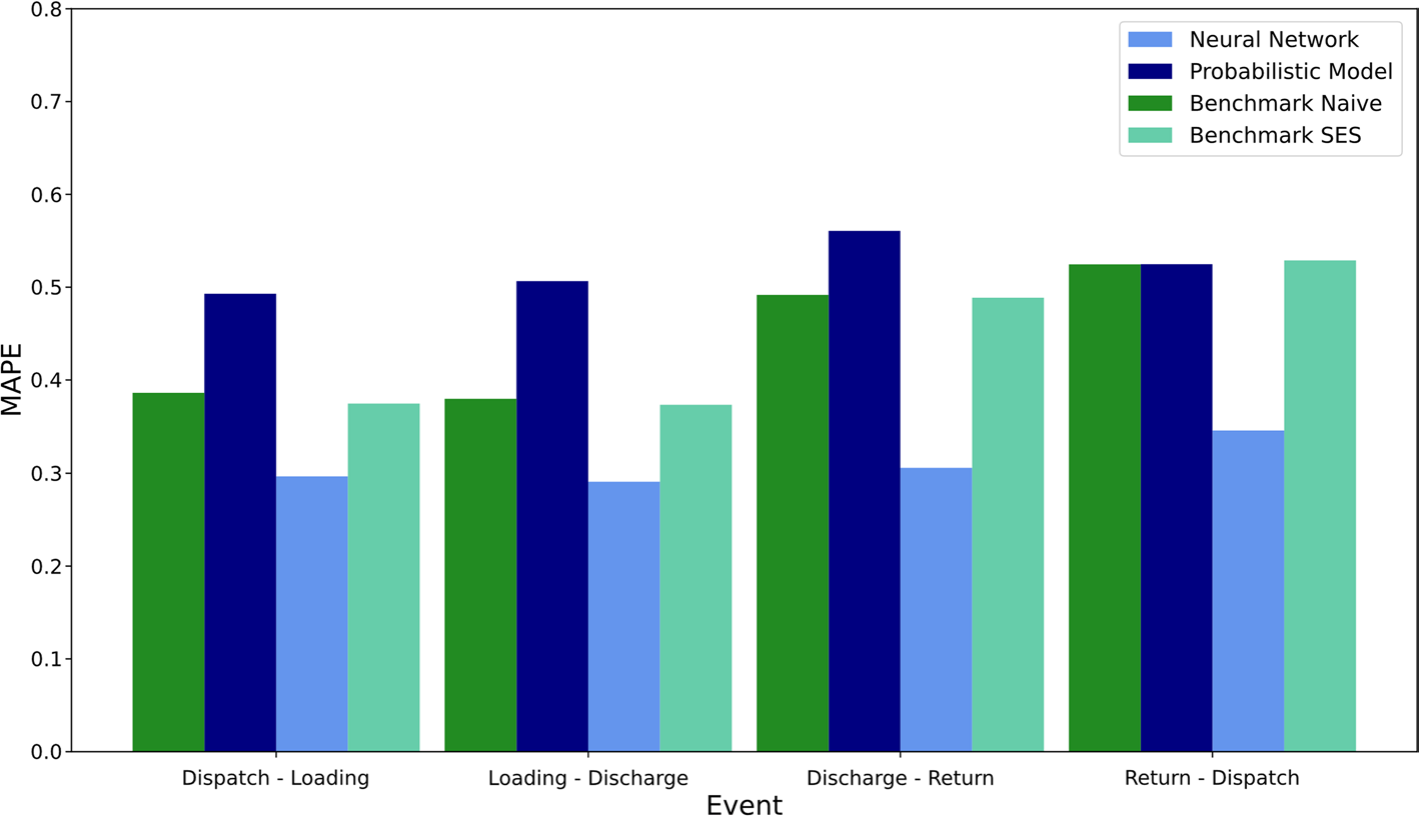
MAPE of the prediction models over the 18 forecasting origins. *Source*: Own figure

In addition to the MAE of the forecasted container availability index over the 18 forecast origins, the mean absolute percentage error (MAPE) was used to quantify the ability of four models to forecast the absolute number of containers dispatched and returned at the locations around the world. The MAPE indicates the relative difference between one event type’s absolute and predicted number of events (dispatch, loading, discharge, return). For example, if 10 containers are dispatched from a container depot in one week at a specific location and 20 containers at another, and the model forecasts 12 dispatches for the first location and 15 for the second location, the absolute percentage error of the first location corresponds to $\frac{|12-10|}{10}=0.2,$ and the absolute percentage error of the second location equals $\frac{|15-20|}{20}=0.25$. Hence, the MAPE of the dispatches in this example is $\frac{0.2+0.25}{2}=0.225$. Figure 12 shows the MAPE of the four models for each of the four event types over the 18 forecasting origins for locations with more than 50 weekly events.

It can be seen in Fig. 12 that the neural network approach outperforms the other three prediction approaches regarding the prediction of the absolute number of container events at the locations in the evaluation set with a MAPE between $0.2909$ and $0.3460$, followed by Benchmark Naïve with a MAPE between $0.3797$ and $0.5246$. However, the probabilistic model is last for three of the four event types with a MAPE between $0.4929$ and $0.5606$. Even though the neural network performs better than the other three models, the MAPE of all models is still relatively high: a MAPE of approximately 0.3 for the neural network approach indicates that the model either over or underestimates the absolute number of container events at the locations by $30\%$. Similarly, the observation was made for the container availability index that the forecasts improve for locations with more data.

## Discussion of results

Related literature in the area of container availability forecasts has focused on predicting the future development of container throughput in specific locations to derive information about the availability of empty containers. This article presented a novel prediction approach that derives information about container availability from estimations of future movements of many individual containers. The advantage of the proposed approach is that the current state of the global container fleet is considered during prediction. This enables the model to anticipate short- and mid-term effects on the container availability’s future development, like the implications of container shortages or temporal closures of specific ports. It was shown in Sect. Results that the novel prediction approach can provide more accurate forecasts than predictions made at the port-level. However, there are two challenges related to the proposed prediction approach. First, a huge amount of data is required to train the model robustly. Second, the processing time tends to be significantly longer since the utilised data is of higher granularity than the input of conventional prediction models.

Most related studies are characterized by one or more of the following aspects:

- relatively small data volume, which prevents training robust models,
- high degree of temporal aggregation (e.g. yearly or quarterly throughput),
- no differentiation of container types, and
- predictions that cover only small regions or even individual ports.

A machine learning model able to forecast the supply and demand of empty containers for different types of containers on a weekly basis was trained enabled by the data corpus consisting of more than 100 million transport events. The utilised data set made it possible to derive container availability for more than 280 locations worldwide. In addition, the concept of the model permits to predict the container movements for an even higher number of individual ports and regions. However, not enough data was available in the data set, especially for a port with a lower container throughput.

Even though it was shown that the proposed approach implemented with feed-forward neural networks and mixture density networks is able to outperform port-based models, the accuracy of the forecasts can be improved even further. It was shown that the quality of the predictions increases with the amount of data available for a location, cf. Fig. 11. It is expected that this trend continues if a larger share of the ports’ container throughput can be used for training and validation.

Especially if the absolute number of containers is utilised for evaluation instead of a container availability index, it is desired to improve the quality of the forecasts. We expect that the discrepancy between the absolute container throughputs’ forecasts and the actual amount is mainly explained by two factors. First, both the training and validation data are influenced by several irregularities, including the covid-19 pandemic, temporal closures of ports, consumption changes, and the general reduction of the global container fleet’s size. Second, a not negligible number of containers is not continuously tracked during the data collection process; thus, several movements of containers are not included in the database – especially over the first months covered. Since machine learning models learn patterns of historical events and forecast them for the future, the models struggle with changes in the data quality as well as shifts in global trade relations. Hence, we argue that the models' forecasts would be even more accurate if the utilised data is more consistent and contains fewer outliers. This statement will be verified in future research.

### Practical implications

A model able to forecast container availability could be used as decision support for various stakeholders in the maritime industry. In addition, such a model could be included as an additional software service in existing applications. Some potential valuable application scenarios are listed in the following:

- Liner shipping and leasing companies could utilise the information provided by the service as decision support for the timely planning and realization of container relocations to or from locations if a deficit or a surplus is foreseeable.
- Leasing and transport companies could apply dynamic pricing to adjust transport prices based on expected market developments to maximise their profits.
- Liner shipping companies could plan transport processes by considering the predicted future container availability to minimise transport costs.
- Terminal operators could utilise container availability forecasts to optimise the planning of their capacities and resources.

**Fig. 13:**
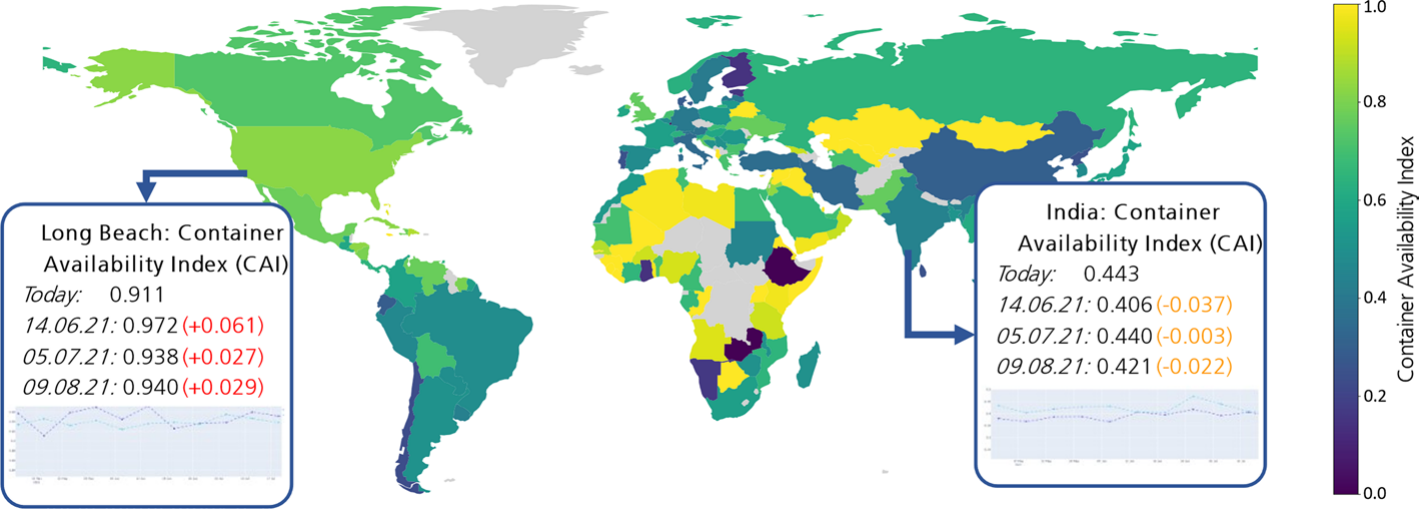
Forecasts of the container availability index on 7th June 2021 made on 9th May 2021 for forty-foot containers. *Source*: Own figure

In addition to the visualisation of the container availability forecasts of ports with individual charts, cf. Figs. 9 and 10, a visualisation of the actual or predicted global container availability could be valuable for decision-makers, cf. Fig. 13. Such a depiction enables users to quickly obtain an overview of surplus and deficit regions, analyze future developments, and make empty container disposition decisions.

### Academic implications

It was shown in this article that forecasts on the container level could yield better prediction quality compared to port-based predictions. The proposed prediction process offers advantages over conventional methods, especially for short- and mid-term forecasts. Both approaches – on container-level and port-based forecasts – are expected to result in similar performance if long-term developments (more than one year) are investigated due to the probabilistic nature of both approaches. Even though the proposed approach's overall prediction quality is superior to conventional methods, it comes at the cost of higher computational requirements.

Future research could focus on further improving the prediction quality of the duration and location prediction models. For example, the use of additional characteristic features, e.g. financial indicators or time-related information, is expected to improve the prediction quality of the forecasting approaches. Additionally, a more extensive hyperparameter optimisation, primarily focusing on the distribution used by the mixture density network, might emend the accuracy of the models. Furthermore, it would be interesting to include more individual ports in the training and evaluation process. However, the number of ports that can be used for training and evaluation depends on the availability of sufficient data.

Another avenue for future research is the development of additional measures and evaluation metrics to quantify the supply and demand of empty containers. Such measures could be used as target variables during the evaluation of both conventional prediction approaches and the proposed method and would provide valuable insights into regional container availability.

## Conclusions

Liner shipping companies frequently transport empty containers from import-dominant regions to export-dominant regions to match the local demand for empty containers. These empty container relocations cause significant financial and capacity-related expenses for the carriers. A novel data-driven prediction approach to support the decision-making process related to empty container logistics was presented in this paper. While previous related work estimated the future container throughput on a port level, the container availability is derived from forecasts of movements of individual containers in the presented approach. Thereby, the models can incorporate knowledge of the current situation of the global container fleet when forecasting the container throughput at the port around the world.

Two prediction models implementing the novel prediction approach were introduced and compared to two conventional methods which estimate the container throughput based on historical data on a port level. The neural network model outperforms the other three approaches, which underlines the applicability of the introduced prediction approach. The prediction quality of the probabilistic model instead is similar to the conventional approaches.

The presented approach using neural networks can approximate the container availability for the selected locations with an average MAE of around 0.05. It was shown that predictions tend to be more accurate if more training and evaluation data are available. While the neural network approach can relatively accurately predict a container availability index, all models fail to estimate empty containers’ absolute weekly supply and demand. The forecasts of the best-performing model differ from the actual values by $30\%$ on average. However, the prediction quality is expected to increase with more high-quality data and fewer exceptional circumstances (i.e. COVID pandemic) represented in the training and evaluation data set.

## Data Availability

The data that support the findings of this study are available from xChange Solutions GmbH [xChange Solutions GmbH, Holstenwall 5, 20,355 Hamburg, Germany] but restrictions apply to the availability of these data, which were used under license for the current study, and so are not publicly available. Data are however available from the authors upon reasonable request and with permission of xChange Solutions GmbH.

## Abbreviations

API: Application programming interface
ARIMA: Autoregressive integrated moving average
CAX: Container availability index
C-TIMING: ConTainer availability index made in Germany
ELU: Exponential linear unit
LSTM: Long-short-term-memory neural network
MAE: Mean absolute error
MAPE: Mean absolute percentage error
MSE: Mean squared error
ReLu: Rectified linear unit
xChange: XChange solutions GmbH

## Footnotes

1 https://www.cml.fraunhofer.de/de/forschungsprojekte1/C_TIMING.html

2 https://www.container-xchange.com/container-leasing/

3 According to https://de.statista.com/statistik/daten/studie/28853/umfrage/kapazitaeten-der-containerschiffsflotte-der-groessten-reedereien/ (accessed on 09.09.2021).

4 According to https://www.statista.com/statistics/198206/share-of-leading-container-ship-operators-on-the-world-liner-fleet/ (accessed on 12.08.2021).

5 https://container-xchange.com/features/cax/

6 18 forecasting origins are used since an insufficient data quantity prevented to evaluate the models with forecasting origins before November 2020 while preprocessing criterion 2 did not allow to utilise forecasting origins later than Februar 2021 as the latest events of the data set date back to July 2021.

7 150 days approximately corresponds to twice the time of an average transport process – from one dispatch to the subsequent dispatch of a container.
